# Icatibant, an inhibitor of bradykinin receptor 2, for hereditary angioedema attacks: prospective experimental single-cohort study

**DOI:** 10.1590/1516-3180.2014.1325652

**Published:** 2014-07-22

**Authors:** Regis Albuquerque Campos, Solange Oliveira Rodrigues Valle, Alfeu Tavares França, Elisabete Cordeiro, Faradiba Sarquis Serpa, Yara Ferreira Mello, Teresinha Malheiros, Eliana Toledo, Elie Mansour, Gustavo Fusaro, Anete Sevciovic Grumach

**Affiliations:** I MD, PhD. Associate Professor in the Discipline of Allergy and Immunology, Faculdade de Medicina, Universidade Federal da Bahia (UFB), Salvador, Bahia, Brazil; II MD, PhD. Attending Physician in the Allergy Outpatient Group, Faculdade de Medicina, Universidade Federal do Rio de Janeiro (UFRJ), Rio de Janeiro, Brazil; III MD. Attending Physician in the Recurrent Infections Outpatient Group, Faculdade de Medicina do ABC (FMABC), Santo André, São Paulo, Brazil; IV MD, MSc. Assistant Professor in the Department of Internal Medicine, Escola Superior de Ciências da Santa Casa de Misericórdia de Vitória (EMESCAM), Vitória, Espírito Santo, Brazil; V MD. Attending Physician in the Allergy Service, Hospital Edmundo Vasconcelos, São Paulo, São Paulo, Brazil; VI MD, PhD. Associate Professor in the Department of Pediatrics, Faculdade de Medicina da Universidade de São José do Rio Preto (Famerp), São José do Rio Preto, São Paulo, Brazil; VII MD. Associate Professor in the Department of Pediatrics, Universidade Estadual de Campinas (Unicamp), Campinas, São Paulo, Brazil; VIII MD. Attending Physician in the Department of Pediatrics, Universidade Federal de Minas Gerais (UFMG), Belo Horizonte, Minas Gerais, Brazil; IX MD, PhD. Professor, Attending Physician in the Recurrent Infections Outpatient Group and Responsible for Clinical Immunology Laboratory, Faculdade de Medicina do ABC (FMABC), Santo André, São Paulo, Brazil

**Keywords:** Angioedemas, hereditary, Complement C1 inhibitor protein, Therapy [subheading], Receptors, bradykinin, Bradykinin, Angioedemas hereditários, Proteína inibidora do complemento C1, Terapêutica, Receptores da bradicinina, Bradicinina

## Abstract

**CONTEXT AND OBJECTIVE:**

Hereditary angioedema (HAE) with C1 inhibitor deficiency manifests as recurrent episodes of edema involving the skin, upper respiratory tract and gastrointestinal tract. It can be lethal due to asphyxia. The aim here was to evaluate the response to therapy for these attacks using icatibant, an inhibitor of the bradykinin receptor, which was recently introduced into Brazil.

**DESIGN AND SETTING:**

Prospective experimental single-cohort study on the efficacy and safety of icatibant for HAE patients.

**METHODS:**

Patients with a confirmed HAE diagnosis were enrolled according to symptoms and regardless of the time since onset of the attack. Icatibant was administered in accordance with the protocol that has been approved in Brazil. Symptom severity was assessed continuously and adverse events were monitored.

**RESULTS:**

24 attacks in 20 HAE patients were treated (female/male 19:1; 19-55 years; median 29 years of age). The symptoms were: subcutaneous edema (22/24); abdominal pain (15/24) and upper airway obstruction (10/24). The time taken until onset of relief was: 5-10 minutes (5/24; 20.8%); 10-20 (5/24; 20.8%); 20-30 (8/24; 33.4%); 30-60 (5/24; 20.8%); and 2 hours (1/24; 4.3%). The time taken for complete resolution of symptoms ranged from 4.3 to 33.4 hours. Adverse effects were only reported at injection sites. Mild to moderate erythema and/or feelings of burning were reported by 15/24 patients, itching by 3 and no adverse effects in 6.

**CONCLUSION:**

HAE type I patients who received icatibant responded promptly; most achieved improved symptom severity within 30 minutes. Local adverse events occurred in 75% of the patients.

## Abstract (pt)

**CONTEXTO E OBJETIVO:**

O angioedema hereditário (AEH) com deficiência de inibidor de C1 manifesta-se por episódios recorrentes de edema envolvendo pele, trato respiratório superior e gastrointestinal. Pode ser letal por asfixia. O objetivo foi avaliar a resposta à terapia dos ataques com icatibanto, inibidor do receptor de bradicinina, recentemente introduzido no Brasil.

**TIPO DE ESTUDO E LOCAL:**

Estudo experimental prospectivo de coorte, sem grupo controle, da eficácia e segurança do icatibanto em paciente com AEH.

**MÉTODOS:**

Pacientes com diagnóstico confirmado de AEH foram incluídos de acordo com os sintomas, independentemente do tempo de início do ataque. Icatibanto foi administrado segundo protocolo aprovado no Brasil. A gravidade do sintoma foi estabelecida continuamente e os eventos adversos foram monitorados.

**RESULTADOS:**

24 ataques em 20 pacientes com AEH foram tratados (19 F:1 M; 19-55 anos; mediana 29 anos). Os sintomas foram: edema subcutâneo (22/24), dor abdominal (15/24) e obstrução de vias aéreas superiores (10/24). O tempo para o início do alívio foi: 5-10 minutos, 5/24 (20,8%); 10-20, 5/24 (20,8%); 20-30, 8/24 (33,4%); 30-60, 5/24 (20,8%) e 2 horas, 1/24 (4,3%). O tempo para a resolução completa variou de 4,3-33,4 horas. Somente efeitos adversos nos locais das injeções foram relatados. Eritema leve a moderado e/ou sensação de ardor foram relatados por 15/24 pacientes, prurido em 3, e 6 não tiveram efeitos adversos.

**CONCLUSÃO:**

Pacientes com AEH tipo I receberam icatibanto com pronta resposta; a maioria teve melhora na gravidade dos sintomas em 30 minutos. Eventos adversos locais ocorreram em 75% dos pacientes.

## INTRODUCTION

Hereditary angioedema (HAE) with C1 inhibitor (C1-INH) deficiency is a rare disease that manifests as recurrent episodes of subcutaneous edema, most commonly involving the skin, upper respiratory tract, oropharynx and gastrointestinal tract. Two forms of HAE have been described: type I HAE with low C1-INH antigenic protein and functional activity (85% of the cases); and type II HAE with normal or elevated protein but low C1-INH function (15% of the cases).^1^ ^,^ ^2^The disease is disabling and can be lethal.^1^ ^,^ ^3^ ^,^ ^4^ No official data exists concerning complications and deaths due to asphyxia among Brazilian HAE patients, but these outcomes have been registered by the Brazilian Association of HAE Patients (ABRANGHE) over recent years (personal communication).

The pathogenesis of edema attacks due to HAE remains elusive. In several reports, bradykinin was found to mediate swelling. Its plasma levels increased during attacks and mice that were deficient in both C1-INH and the bradykinin receptor 2 (BR-2) gene showed diminished vascular permeability,^5^ ^-^ ^7^ thus confirming the previous evidence. Considering the importance of bradykinin binding and activation of BR-2 in angioedema formation in HAE patients, it would be possible for acute attacks to be treated with the BR-2 antagonist, icatibant. 

Effective management of HAE targets either prevention or treatment of attacks.^1^ Drugs for both approaches have been available since the late 1970s, but not uniformly registered.^2^ In Brazil, until icatibant was approved recently, fresh frozen plasma was the only available therapy for acute HAE attacks. 

## OBJECTIVE

The aim of this study was to describe the clinical response of HAE patients to therapy for HAE attacks using icatibant, an inhibitor of the bradykinin receptor, and the possible side effects. This was the first report on the use of this new drug in a real-life setting.

## METHODS

We conducted a prospective experimental single-cohort study on 20 patients who were treated using icatibant. In order to be eligible for treatment in accordance with the Brazilian approval, adult patients needed to have a documented diagnosis of hereditary angioedema (including a low C4 level, a normal C1q level and a low antigenic or functional C1 inhibitor level) and to have a well-documented history at outpatient clinics, as stipulated in the Brazilian guidelines.^1^ The exclusion criteria for treatment were other differential diagnoses of HAE (drugs such as hormones and angiotensin converser enzyme (ACE) inhibitors), pregnancy and age greater than 65 years. Subjects were not required to change any of their regular medications, including androgens or antifibrinolytic drugs. Icatibant was administered during an attack of moderate or severe intensity, involving the abdomen, face or external genitalia, with symptoms of upper airway obstruction such as a change in voice tone and difficulty in swallowing, regardless of the time that had elapsed since the onset of symptoms. The severity of the attacks was established in accordance with Giavina-Bianchi et al.^1^ and Bowen et al.^2^ One syringe (30 mg) of icatibant was slowly administered subcutaneously in the abdominal wall. Brazilian legislation also stipulates that a second injection of the same drug could be administered if necessary, six hours after the first administration.

Prior to icatibant administration, all participants were informed about its effects and potential adverse reactions and gave their consent to treatment. Self-administration is not allowed, according to Brazilian legislation; therefore, all injections were administered under medical supervision, as established by the Brazilian Health Surveillance Agency (Agência Nacional de Vigilância Sanitária, ANVISA), with access to medical emergency facilities. 

The physicians and the patients were asked to describe the symptoms at each site affected (extremities, throat, abdomen, face and external genitalia). Symptom severity was assessed continuously until the subject reported achieving relief. The patients were allowed to leave the hospital facilities after improvement, but were subsequently contacted to confirm that complete resolution of symptoms had occurred. Safety was evaluated by assessing adverse events, changes in physical findings and vital signs before and after the injection.

## RESULTS

**Figure 1:**
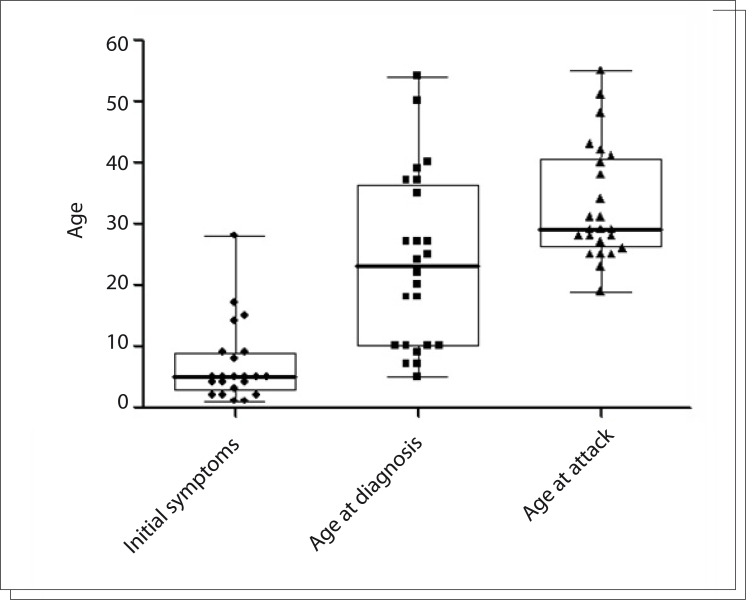
Age at the time of first symptoms, age at diagnosis and icatibant application among hereditary angioedema patients.

From August 2011 to February 2012, 24 attacks were treated using icatibant, in 20 HAE type 1 patients (19 females and one male). One patient was treated four times and another, twice, in different attacks. The median age at the initial appearance of HAE symptoms was 5 years old (range 1-28 years), with median age at diagnosis of 23 years (range 5-54 years). The youngest patient treated with icatibant was 19 years old and the oldest, 55 years old (median 29 years) (Figure 1).

**Figure 2:**
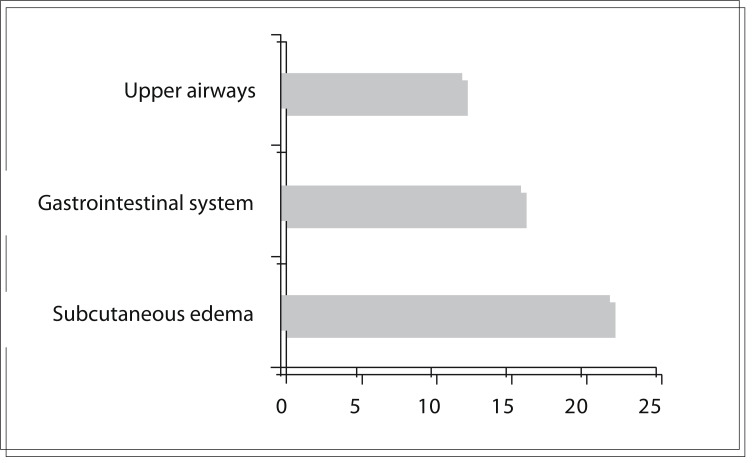
Clinical symptoms presented by hereditary angioedema patients before icatibant application.

The following involvement was observed during the attacks: subcutaneous edema (22/24); gastrointestinal pain (16/24); upper airway obstruction (12/24), reported as changes in voice tone and swallowing difficulty; and laryngeal edema (2/24) (Figure 2). Facial edema was observed in 16/24 and in nine cases was also associated with abdominal pain. Gastrointestinal pain alone was present in 1/24 patients only. In 18 patients, the HAE attack was classified as severe and in six as moderate. At the time of the treatment for the HAE attack, fifteen patients were taking androgen prophylaxis (4/15 using oxandrolone and 11/15 using danazol), one patient was being treated with antifibrinolytics and eight patients were not undergoing any specific therapy.

**Figure 3:**
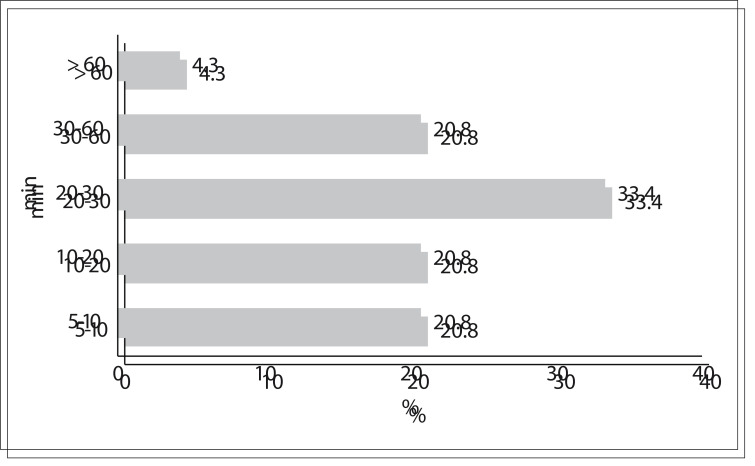
Time taken to achieve initial relief from symptoms during hereditary angioedema attacks.

Administration of icatibant was started at an average of 6.3 hours (median = 6 hours; range 2-12 hours) after the onset of symptoms. The estimated time taken for relief to begin after the injection was: 5-10 minutes (5/24; 20.8%); 10-20 (5/24; 20.8%); 20-30 (8/24; 33.4%); 30-60 (5/24; 20.8%); and two hours (1/24; 4.3%) (Figure 3). The upper airway obstruction improved first, followed by the abdominal pain, whereas the skin swelling took a longer time to resolve. The time taken to achieve complete resolution of symptoms was variable, from 4.3 to 33.4 hours. None of the patients required more than one injection for the same episode; however, two patients presented recurrence within 24 hours but with no medical intervention, and spontaneous regression occurred. Mild to moderate erythema and/or feelings of burning were reported in the cases of 15/24 applications; itching was described after injections in 3 cases; and 6 treatments had no adverse effect. The patient who was treated for four different attacks showed a good clinical response in each instance, and the time from treatment to the resolution of symptoms remained constant.

## DISCUSSION

In this study, we reported on treatment using icatibant during 24 episodes of acute swellings in HAE type I patients in a real-life setting in Brazil. All the patients showed good clinical responses to this treatment and most of them reported clinical relief even before any change was observed in cutaneous swelling. The initial onset of relief was earlier for upper airway obstruction symptoms. Subcutaneous edema was the clinical manifestation that persisted the longest. Most of the patients showed an improvement in respiratory and gastrointestinal symptoms within 30 minutes following the injection. Importantly, total regression of the complaints occurred within 24 hours (median), in comparison with attacks in which there was no specific drug therapy, which lasted three to five days, as previously reported.^8^ ^,^ ^9^

Evaluation of icatibant was performed in three controlled trials: FAST-1, FAST-2 and FAST-3. In the FAST-1 trial, statistical significance between the drug and placebo regarding the time taken to achieve symptom relief was not found, although the patients receiving icatibant reported the first endpoint improvement after 2.5 hours and those with the placebo, after 4.6 hours. The analysis parameters used in this trial and the time taken for the treatment to be introduced probably influenced the results.^8^ ^,^ ^10^ In the second study, the efficacy of icatibant was compared with tranexamic acid and a significant reduction in symptoms was observed (2.0 hours versus 12.0 hours). FAST-3, which was a randomized placebo-controlled study, demonstrated that icatibant was effective in treating HAE attacks in adults (88 subjects; 43 with icatibant and 45 with placebo).^10^

In our series of patients, the upper airway obstruction symptoms were the first to resolve. Greve at al. found the same results after reporting on administration of icatibant in 141 attacks in a single patient.^11^ During the FAST studies, most of the laryngeal attacks were treated as an open-label phase and the median time taken to achieve relief was longer than one hour.^8^ ^,^ ^10^ The discrepancy between the findings from those studies and our results may be due to the different analysis parameters used in those studies. The quick response may have been associated with anatomical issues in the upper airways, in which even a small amount of edema can lead to significant clinical repercussions. Also, this observation points to the importance of mediation of this symptom through bradykinin binding to B2 receptors, which is a leading cause of death in HAE patients. 

Icatibant is approved in Brazil for use under medical supervision only, i.e. patients are not allowed to inject themselves. This resulted in a delay in the therapy with a median elapsed time of 6 hours until application, and a maximum of 12 hours. However, this did not influence the efficacy of the therapy, since our patients who received therapy more than 10 hours after the attack started achieved improvement within the same length of time as shown by those who had early access to medical assistance, as has previously been reported.^12^ Development of home treatment strategies for patients with HAE is an important step towards improving the management of this debilitating condition, which would lead to a better response and, consequently, better quality of life.^12^ ^,^ ^13^ Approval for self-administration is under analysis by the Brazilian authorities.

The attack was classified as severe in approximately 60% of the patients studied (14/24), and suggestive clinical manifestations associated with laryngeal edema were the indication for icatibant use in 50% of the situations in our report. The patients reported that voice changes and facial edema were the sensations that preceded airway obstruction. Although no official classification of severity is available, the Brazilian guidelines was used for therapy indication.^1^ Therefore, the severity of the disease may explain some of the delays in achieving complete improvement that were seen in some individuals in this subgroup of patients. However, patients could choose to have even mild attacks of HAE treated, if the therapy was available. In addition, combination of preventive and on-demand therapy with reduction of the androgen dose and treatment of breakthrough attacks using C1 inhibitor or icatibant has been proposed.^14^ ^,^ ^15^ Such observations are limited by the type of study developed, with no control group.

Two patients who received icatibant more than once did not observe any reduction in the efficacy of the therapy and did not present any increase in adverse events. Greve et al. reported that icatibant was used 141 times in one patient without any loss of efficacy.^11^ There were no adverse events in one third of our cases and the complaints reported consisted of local burning, hyperemia and mild itching. These symptoms were self-limited and related to local release of histamine induced by subcutaneous injection of icatibant, a development that may be attenuated by H1-antihistamines.^16^

Sixty percent (12/20) of the patients who received icatibant were undergoing prophylactic treatment with androgens. In fact, the approved therapy for HAE within the public healthcare system in Brazil only includes danazol. It has been estimated that about 25% and 50% of the HAE patients in Switzerland and Austria, respectively, receive long-term therapy with androgens, with variable indications.^15^ Long-term prophylaxis with attenuated androgens is effective in many patients, but side effects such as virilization in females, weight gain and other factors result in restrictions on their use. It is possible that restricted access to therapy probably leads physicians to introduce prolonged use of drugs as well as higher androgen doses in HAE patients.

Brazil was the only South American country with access to icatibant at the time of reporting this experience. The health authorities in Colombia and Argentina have approved icatibant, but the drug is not available yet.^17^ Regarding diagnosis and therapy of HAE in Latin America, we are at the forefront of a process that is just beginning,^18^ except in Argentina, where the first reports were published almost 30 years ago and plasma-derived C1 inhibitor is available.^17^ Icatibant was launched three years ago in Brazil and the use of this drug is not financially supported by the government, although some health insurance companies reimburse its use on demand. 

Most of the patients (65%) reported their first symptoms when they were five years old; only four of them presented after adolescence. The median age for diagnosis was 23 years, thus suggesting that there is a delay in identifying HAE patients. One patient was 55 years old by the time that the disease was recognized, but its symptoms first presented when she was 15 years old; her case is a testament to how restricted knowledge about HAE is. If we consider the fact that there is almost no registration of cases in other Latin American countries, it becomes obvious that many opportunities to treat HAE patients have been lost. Gastrointestinal complaints were reported by 62.5% of our patients and were not associated with facial edema in 21% of the cases treated in our experience.

Marqués et al. reported that one potential future use for icatibant would be for short-term prophylactic HAE treatment, and they reported on its use for a patient with previous severe attacks during some procedures.^19^ The patients who may benefit from switching to on-demand therapy, or from combining preventive and on-demand therapy, include women, children and adolescents, as well as patients with various risk factors for androgens such as organ toxicity, cardiovascular disease or possible drug interactions.^20^ Use of icatibant has been restricted for patients under 18 years of age and for patients with cardiac diseases.^21^

Medication cost is a major concern for both patients and physicians. The US Hereditary Angioedema Association has estimated that hospital stays and emergency department visits during acute attacks comprise 68% of the cost of a severe attack.^22^As understanding of HAE physiopathology and management improves, decisions regarding continuous or on-demand therapy should become easier, and studies evaluating pharmacoeconomics will certainly help. 

## CONCLUSIONS

The HAE type I patients who received icatibant responded promptly; most of them achieved improved symptom severity within 30 minutes. Mild local adverse events were reported in 75% of the patients. The introduction of this new drug has opened up new perspectives for HAE patients regarding therapies for attacks.
